# Phagolysosomal Survival Enables Non-lytic Hyphal Escape and Ramification Through Lung Epithelium During *Aspergillus fumigatus* Infection

**DOI:** 10.3389/fmicb.2020.01955

**Published:** 2020-08-20

**Authors:** Constanze Seidel, Sergio D. Moreno-Velásquez, Nagwa Ben-Ghazzi, Sara Gago, Nick D. Read, Paul Bowyer

**Affiliations:** Manchester Fungal Infection Group, Faculty of Biology, Medicine and Health, The University of Manchester, Manchester Academic Health Science Centre, Core Technology Facility, Manchester, United Kingdom

**Keywords:** *Aspergillus fumigatus*, epithelial cell infection, membrane compartmentalisation, aspergillosis, phagolysosome, airway epithelium, microscopy

## Abstract

*Aspergillus fumigatus* is the most important mould pathogen in immunosuppressed patients. Suboptimal clearance of inhaled spores results in the colonisation of the lung airways by invasive hyphae. The first point of contact between *A. fumigatus* and the host is the lung epithelium. *In vitro* and *ex vivo* studies have characterised critical aspects of the interaction of invasive hyphae on the surface of epithelial cells. However, the cellular interplay between internalised *A. fumigatus* and the lung epithelium remains largely unexplored. Here, we use high-resolution live-cell confocal microscopy, 3D rendered imaging and transmission electron microscopy to define the development of *A. fumigatus* after lung epithelium internalisation *in vitro*. Germination, morphology and growth of *A. fumigatus* were significantly impaired upon internalisation by alveolar (A549) and bronchial (16HBE) lung epithelial cells compared to those growing on the host surface. Internalised spores and germlings were surrounded by the host phagolysosome membrane. Sixty per cent of the phagosomes containing germlings were not acidified at 24 h post infection allowing hyphal development. During escape, the phagolysosomal membrane was not ruptured but likely fused to host plasma membrane allowing hyphal exit from the intact host cell in an non-lytic Manner. Subsequently, escaping hyphae elongated between or through adjacent epithelial lung cells without penetration of the host cytoplasm. Hyphal tips penetrating new epithelial cells were surrounded by the recipient cell plasma membrane. Altogether, our results suggest cells of lung epithelium survive fungal penetration because the phagolysosomal and plasma membranes are never breached and that conversely, fungal spores survive due to phagosome maturation failure. Consequently, fungal hyphae can grow through the epithelial cell layer without directly damaging the host. These processes likely prevent the activation of downstream immune responses alongside limiting the access of professional phagocytes to the invading fungal hypha. Further research is needed to investigate if these events also occur during penetration of fungi in endothelial cells, fibroblasts and other cell types.

## Introduction

Invasive fungal infections are a major cause of mortality, with more people dying from the ten main invasive fungal diseases than from tuberculosis or malaria (Brown et al., 2012). Despite the development of new antifungal drugs and medical procedures, mortality rates for these diseases remain extremely high (Bongomin et al., 2017). This is the case of fungal lung infections caused by the opportunistic mould *Aspergillus fumigatus* for which mortality can reach 90% despite treatment. *A*. *fumigatus* is a saprophytic mould which produces millions of small conidia (2–3 μm) that are released into nearly all human accessible habitats (Bennett, 2010; O’Gorman, 2011; Kwon-Chung and Sugui, 2013; Knox et al., 2016). Typically, humans inhale up to several hundred *A. fumigatus* conidia per day, which are efficiently eliminated by the innate lung defences (Mullins and Seaton, 1978; Latgé, 1999; Balloy and Chignard, 2009). However, in some immunocompromised patients or those with a prior respiratory condition such as a past history of tuberculosis infection, COPD, asthma or cystic fibrosis, conidia can evade the host response, germinate and colonise the lung epithelium leading to the development fungal disease (Wasylnka et al., 2005; Mccormick et al., 2010; Kosmidis and Denning, 2015; van de Veerdonk et al., 2017; Gago et al., 2018).

The respiratory epithelium is the initial point of contact of inhaled conidia with the host (Filler and Sheppard, 2006). Although professional phagocytes, like alveolar macrophages, have been traditionally described as the main host effectors in the anti-*Aspergillus* response, an increasing body of evidence suggests the airway epithelium is an extension of the innate immune system and plays a critical role in fungal clearance (Herzog et al., 2008; Osherov, 2012; Bertuzzi et al., 2019; Amich et al., 2020). Additionally, uptake of *A. fumigatus* by epithelial cells triggers the activation of signalling pathways leading to the release of cytokines and antimicrobial peptides facilitating a coordinated immune response (Wasylnka and Moore, 2002; Bellanger et al., 2009; Sharon et al., 2011; Escobar et al., 2016; Richard et al., 2018).

During its contact with the airway epithelium, conidia have been shown to adhere to the epithelial cells and extracellular matrix (Bromley and Donaldson, 1996; DeHart et al., 1997; Latgé, 1999; Bertuzzi et al., 2019). Invasion of the lung epithelia is a common pathogenic strategy used by microorganisms to entry into the vascular endothelium and cause a systemic infection (DeHart et al., 1997; Paris et al., 1997; Han et al., 2011; Sun et al., 2012; Bertuzzi et al., 2019). Several *in vitro* and *ex vivo* infection studies have shown that bronchial and alveolar epithelial cells can internalise adherent fungal conidia in a time-dependent manner (Wasylnka and Moore, 2002; Han et al., 2011; Xu et al., 2012; Bertuzzi et al., 2014; Gago et al., 2018; Richard et al., 2018; Clark et al., 2019). *In vitro* analyses of the interaction between lung epithelial cells and *A. fumigatus* have shown that epithelial cells are able to form pseudopods to facilitate conidia engulfment on an actin, cofilin-1, phospholipase-D-dependent manner (Paris et al., 1997; Wasylnka and Moore, 2002; Han et al., 2011; Bertuzzi et al., 2014; Bao et al., 2015). Subsequently, internalised conidia are trafficked into acidic phagosomes, where class III PI3P kinases are critical for processing (Clark et al., 2019). This process strongly limits viability of internalised conidia and only 3% survive after 24 h (Wasylnka and Moore, 2003). This small proportion of conidia can germinate and escape the epithelial cells without clear evidence of host death (Amin et al., 2014; Bertuzzi et al., 2014). However, the spatial and temporal interplay between the host and internalised *A. fumigatus* spores upon escape have not been explored.

Here, we studied the dynamics involved in the early stages of the growth of *A. fumigatus* during infection of alveolar and bronchial epithelial cells on a time-dependent manner. Our data demonstrate that survival of the lung epithelium upon infection relies on the phagolysosomal and plasma membrane compartmentalisation of internalised *A. fumigatus* thus limiting direct fungus-host contact.

## Materials and Methods

### *A. fumigatu*s Strains and Growth Conditions

*Aspergillus fumigatus* A1163 expressing cytosolic GFP and RFP under the control of the *B-tubulin* gene were kindly provided by Dr. Michael Bromley (Macdonald et al., 2019). *A. fumigatus* ATCC 46645 expressing cytosolic YFP [PgpdA::*yfp*(ptrA)] constructed as previously described (Lother et al., 2014) was a kind gift from Prof. Sven Krappmann, and used for hyphal extension imaging. Strains were maintained at 37°C for 5 days in *Aspergillus* minimal media (AMM) containing 2% sucrose, 1.5% agar (w/v). Conidia were harvested with 0.05% PBS Tween 20 and concentration was determined by using a haemocytometer.

### Cell Lines

The type II alveolar epithelial cell line A549 (Lieber et al., 1976; American Type Culture Collection CCL-18) and the cytosolic-GFP stable A549 cell line (SC043-Bsd, Amsbio, United States) were maintained by serial passage in Dulbecco’s Modified Eagle Medium (DMEM) supplemented with 10% foetal bovine serum (FBS), streptomycin (100 μg/ml) and penicillin (16 μg/ml). 16HBE bronchial epithelial cells (Cozens et al., 1994) were a kind gift from Dr. Sarah Herrick (The University of Manchester). 16HBE cells were maintained in Eagle Minimal Essential Media (MEM) supplemented with L-glutamine (1%), FBS (10%), streptomycin (100 μg/ml) and penicillin (16 μg/ml). All cell lines were grown at 37°C, 5% CO_2_ and used after the second or third passage. All reagents were purchased from Sigma unless otherwise stated.

### Exposure of Epithelial Cells to *A. fumigatus* Conidia

For all experiments, 10^5^ epithelial cells/ml were seeded in 2- or 8-well chambers (Nunc LabTek chamber slide system, Life Technologies) and incubated at 37°C, 5% CO_2_ for 24 h to approximately 90% confluency. Monolayers were then challenged with 10^6^ *A. fumigatus* spores/ml (1:10 multiplicity of infection) from 6 to 24 h depending on the experiment’s readout. Epithelial cells were washed 3 times with pre-warmed PBS and cells were incubated with pre-warmed DMEM/F12 medium (without phenol red) for live-cell imaging.

### Characterisation of *A. fumigatus* Development Within Lung Epithelial Cells

To track the cell biology of internalised *A. fumigatus* spores, the cytoplasmic membrane of the 16HBE and A549 epithelial monolayers was either stained using CellMask Deep Red Membrane dye (Life Technologies, Eugene, OR, United States) or transiently transfected using the CellLight^™^ Plasma Membrane-GFP BacMam 2.0 system (GFP-myristolyation/palmitoylation sequence from LcK tyrosine kinase) (Life Technologies, United States) (Celik et al., 2013). For CellMask Deep Red staining (excitation = 649 nm, emission = 666 nm), epithelial cells were stained after infection for 10 min at 37°C and 5% CO_2_ using a final concentration of 5 μg/ml of the dye. To induce transient expression of GFP in the host plasma membrane, epithelial cell monolayers at a confluency not higher than 70% were transfected using the CellLight^™^ Plasma Membrane-GFP system (excitation = 488 nm, emission = 510 nm) according to manufacturer’s instructions; cells were then incubated until confluence and further challenged with *A. fumigatus* spores as described above. Transfection efficiency was below 100%.

For the detection of acidic intracellular compartments, infected monolayers were stained with 10 nM of LysoTracker DND 99 red (excitation = 577 nm, emission = 590 nm; Life Technologies, Waltham, MA, United States). Additionally, phagolysosomal membranes were labelled by transient expression of the lysosomal associated membrane protein (LAMP-1) using the CellLight^™^ Lysosome-GFP or RFP BacMam 2.0 system (Life Technologies, United States) on uninfected and subconfluent monolayers as described above. After staining with any of the dyes, monolayers were washed twice with pre-warmed PBS and maintained in supplemented DMEM.

All the experiments aiming to characterise the intracellular events of *A. fumigatus* interaction with the lung epithelium were done using A549 alveolar epithelial cells. Experiments aiming to characterise the mechanistic of *A. fumigatus* fate and escape during the infection of the lung epithelia and phagosome maturation were also performed using 16HBE bronchial epithelial cells.

### Nystatin Protection and Cell Viability Assays

The nystatin protection assay was performed as previously described (Wasylnka and Moore, 2002). Briefly, confluent epithelial monolayers were challenged with 10^6^ spores of *A. fumigatus* either expressing GFP or RFP cytosolic proteins for 4 h. Monolayers were then washed twice with pre-warmed PBS and incubated with 50 μg/ml of nystatin in cell culture media (DMEM or MEM for A549 or 16HBE, respectively), at 37°C, 5% CO_2_ for 3 h. Samples were washed twice with pre-warmed PBS and further incubated for 1 h in supplemented DMEM/MEM until analysis. Susceptibility of the strains to nystatin was verified as previously reported (Bertuzzi et al., 2014). Live-cell imaging was performed using phenol red-free DMEM/F12 Medium (Thermo Fisher Scientific) supplemented with 10% FBS, 15 mM HEPES (pH 7).

### Confocal Microscopy

Live-cell imaging at high resolution was performed using a laser scanning confocal microscope (Leica, TCS SP8 X), which was mounted on an inverted microscope and equipped with photo multiplier tubes (PMT), hybrid GaAsP (HyD) detectors and a 63× water immersion objective. A Leica turntable white light laser (WLL, 450-750 nm), argon laser (458, 476, 488, and 496 nm) and UV laser (405 nm) were used for fluorescence excitation. Simultaneous brightfield images were captured with a transmitted light detector. The laser intensity and exposure of the cells were kept to a minimum to reduce photobleaching and phototoxic effects. A 40x/0.85 NA dry and a 63x/1.4 NA water objective lens were used. Live-cell imaging was carried out at 37°C and 5% CO_2_ in a microscope-controlled chamber (Cube & Box, Switzerland). Confocal images were captured using Leica microsystem CMS software (v. 3.3, LAS AF). To visualize the GFP or RFP targeted to the cytoplasm of *A. fumigatus*, excitation of fluorescent proteins was performed at 488 and 555 nm, respectively. The fluorescent signal emitted was detected over the range of 505 to 550 nm for GFP, and 570 to 700 nm for RFP. To image the host plasma membrane and acidic compartments, the dyes CellMask deep red (Excitation/Emission 649/660-750 nm), LysoTracker^™^ red DND-99 (Excitation/Emission 577/590-700 nm), CellLight^™^ Lysosomes-RFP BacMam 2.0 (Excitation/Emission 555-560/570-590 nm) and CellLight^™^ Plasma membrane-GFP BacMam 2.0 (Excitation/Emission 485-488/500-550 nm) were used. 3D images and 4D videos of vertical cross sections through infected monolayers were generated using the IMARIS software package (Bitplane AG, Zurich, Switzerland). IMARIS images were then processed using ImageJ (v. 1.44, MacBiophotonics) and Adobe Photoshop (v. 13, Adobe). Live-cell imaging videos generated in this study were exported from LAS AF software and processed in FIJI^1^.

For quantitative data analysis of the conidia area, confocal images were segmented and the surface area was measured in IMARIS; length of the main hyphae was estimated using the NeuronJ plugin in FIJI as previously reported (Shen et al., 2016). The percentage of *A. fumigatus* spore germination and hyphal length inside or outside the epithelial monolayer were determined in a minimum of 500 events from experiments run in biological and technical triplicates using ImageJ. The percentage of conidia internalisation was estimated as described in Bleichrodt et al. (2020). The fluorescence intensity within acidified phagolysosomes or non-acidified phagolysosomes was determined in arbitrary fluorescence units using IMARIS.

### Electron Microscopy

Correlative live-cell imaging (confocal microscopy) and Transmission Electron Microscopy (TEM) was performed to secure imaging of cells in the desired stage of infection. For that, A549 alveolar epithelial cells were seeded on 35 mm glass bottom petri dishes with a grid (MatTek, Ashland, MA, United States) until 40% confluence and consequently infected with GFP-expressing *A. fumigatus* strain for 12 h. Cells were treated with nystatin at 4 h post-infection as described above. To establish the coordinates of the areas of interest for comparative confocal-electron microscopy analyses, live-cell 3D datasets were generated using confocal laser microscopy. Samples were then fixed with 4% formaldehyde and 2.5% glutaraldehyde in 0.1 M HEPES buffer (pH 7.2). Subsequently, fixed samples were treated with 1% osmium tetroxide and 1.5% potassium ferrocynaide in 0.1 M cacodylate buffer (pH 7.2) for 1 h and then, treated with 1% uranyl acetate in water for an extra hour. Samples were dehydrated in increasing ethanol series, infiltrated with TAAB 812 resin and polymerized for 24 h at 60°C. The coordinates of the areas of interest in resin blocks were identified using the replica of etched location grid from MatTek coverslips. Serial ultrathin sections were cut with Reichert Ultracut ultramicrotome and observed with FEI Tecnai 12 Biotwin microscope at 100 kV accelerating voltage. Images were taken with Gatan Orius SC1000 CCD camera in the Electron Microscopy Core Facility at the University of Manchester.

### Statistical Analyses

Statistical analysis was performed using IBM SPSS software (version 20.0) and GraphPad Prism v8.1 (La Jolla, CA, United States). Statistical analysis included two-tailed Student’s *t*-tests for comparing pairs of normally distributed data, one-way ANOVA with Tukey’s *post hoc* test for comparing datasets containing multiple treatment groups and two-way ANOVA for comparing differences in germination time across treatments. Differences in proportions were analysed using Fisher’s exact test. Comparisons were deemed significant with a *P-*value of ≤0.05. All experiments were performed in biological and technical triplicates. Data is represented as mean and standard deviation, unless other stated.

## Results

### Morphogenesis of *Aspergillus fumigatus* During Early Stages of the Lung Epithelia Infection

**FIGURE 1:**
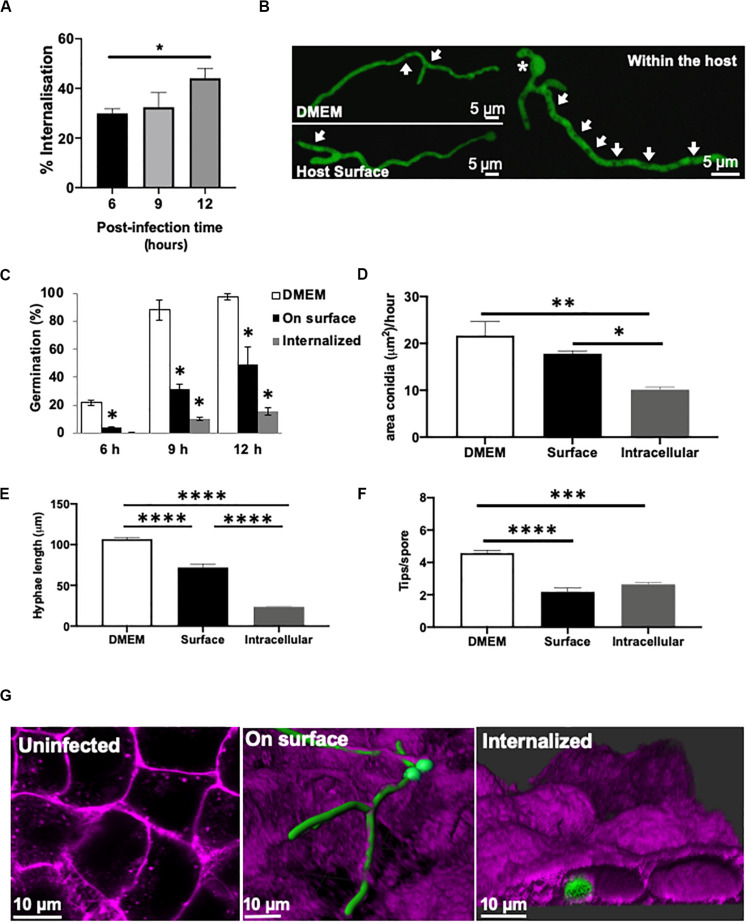
Germination and morphology of internalised *A. fumigatus* is impaired during infection of type II alveolar cells. **(A)** *A. fumigatus* spore uptake by A549 epithelial cells. **(B)** Live-cell imaging of hyphae of *A. fumigatus* expressing cytoplasmic GFP in DMEM, on the alveolar host surface or within the host cells at 37°C, 5% CO_2_ for 18 h in the absence of nystatin. Arrows indicate vacuoles. Asterisks highlight sites of second germ tube initiation. *A. fumigatus* **(C)** germination rate (%), **(D)** swelling rate (%), **(E)** hyphal extension rate and **(F)** degree of branching in the presence or absence of A549 alveolar cells **(G)** Live-cell imaging of the different strategies of *A. fumigatus* infections of A549 alveolar cells. Magenta = host’s plasma membrane stained with CellMask deep red. Green = *A. fumigatus* expressing cytosolic GFP. Measurements were performed in three biological and technical replicates (average ± standard deviation [SD]). ^∗^*P* < 0.05; ^∗∗^*P* < 0.01; ^∗∗∗^*P* < 0.001; ^****^P < 0.0001.

The adhesion and internalisation of *A. fumigatus* conidia by alveolar epithelial cells has previously been described (Wasylnka and Moore, 2002, 2003; Bertuzzi et al., 2014). However, little is known about fungal development and host cell dynamics of internalised *A. fumigatus* spores during lung epithelia infection. To address this question, the isotropic growth (increment on the spore area overtime after breaking dormancy) and germination (emergence of a germ tube with polarised growth) of fluorescent labelled *A. fumigatus* spores within and on the lung epithelia surface was comparatively analysed in a 12 h infection experiment. This time point was chosen as spore uptake was gradually increased on a time-dependent manner (Figure 1A). Isotropic growth rates in culture media were used as control.

Germination and the rate of area increment for swelling spores were significantly higher in culture media (12 h percentage of germination = 98%; swelling rate = 20.66 μm/h) compared to spores growing on (12 h percentage of germination = 57.8%; swelling rate = 17.8 μm/h) or even inside the host cells (12 h percentage of germination = 11.5%; swelling rate = 10.28 μm/h) (Figures 1B–D). Interestingly, *A. fumigatus* germlings growing on the epithelia surface showed a significant decrease in hyphal length, decreased spore swelling and increased hyphal tip branching when compared to those incubated the absence of host cells (*P* < 0.05) (Figures 1D–F). After 20 h of *A. fumigatus-*A549 co-culture, hyphae on the epithelial surface developed into an extensive fungal network that covered the monolayer’s surface (Figure 1G). This *A. fumigatus* growth pattern was similar to that shown by *A. fumigatus* spores growing in the absence of epithelial cells (Figure 1A).

In order to monitor the growth and germination of exclusively internalised *A. fumigatus* spores during early infection stages, A549 epithelial cells were treated with nystatin. Spore swelling and growth was significantly reduced in those germinating internalised spores (Figures 1C,D and Supplementary Video 1). The formation of a second germ tube opposite to the first germ tube was frequently observed after germination of internalised spores (Figure 1B). Branching of internalised germlings (2.64 ± 0.21 hyphal tips/spore) and in those attached to the host surface (2.18 ± 0.42 hyphal tips/spore) was significantly lower than for germlings growing in the absence of cells (4.58 ± 0.30 hyphal tips/spore), (*P* < 0.05) (Figure 1F). This branching pattern occurred rarely in *A. fumigatus* germlings growing in cell culture media. Furthermore, an increased number of vacuoles was observed in internalised *A. fumigatus* germlings than those growing on the surface of the lung epithelia (Figure 1B). Maximal-3D Z-stack projections revealed that during early infection with *A. fumigatus*, the cellular integrity of the epithelial monolayer remained apparently unaffected (Supplementary Video 2).

In order to confirm these observations in a different cellular system, *A. fumigatus* fate was analysed during its interaction with 16HBE bronchial epithelial cells. A different cellular morphology and growth pattern of *A. fumigatus* during infection characterised by increased extracellular and decreased intracellular swelling and impaired intracellular branching was observed compared to alveolar epithelial cells (Supplementary Figure 1). Overall, internalised *A. fumigatus* conidia showed delayed germination (Supplementary Figure 1F), impaired growth and morphogenesis in both, alveolar and bronchial epithelial cell lines.

### Internalised *A. fumigatus* Can Grow Between Adjacent Epithelial Cells

**FIGURE 2:**
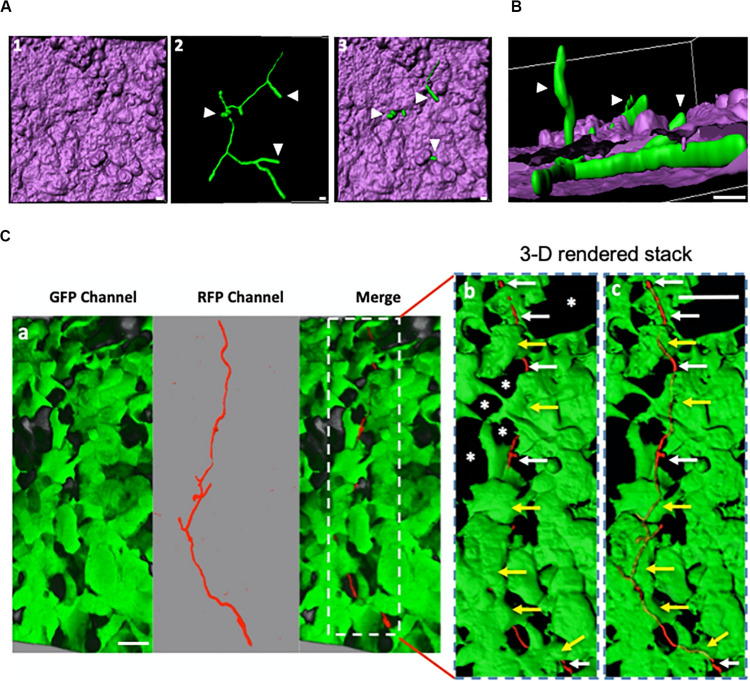
Internalised *A. fumigatus* can expand between adjacent epithelial cells. **(A)** Live-cell imaging of hyphae of *A. fumigatus* cytoplasmically labelled with GFP infecting a monolayer of A549 alveolar cells cultured in DMEM at 37°C, 5% CO_2_ for 18 h. 1- 3-D rendered image of an infected alveolar monolayer stained with Cell Mask Deep Red. 2- Internalised *A. fumigatus* hyphae 3- Merge of 1 and 2. Scale bars: 5 μm. **(B)** Lateral view of the 3-D confocal stack displayed in **(A)** Arrowheads indicate hyphal tips escaping the host monolayer Scale bar: 5 μm. **(C)** *A. fumigatus* hyphal (red) invasion of the epithelial cells (green) 18 h post-inoculation, a magnification of the part indicated with dashed white rectangle with surface rendering, and with 60% transparency demonstrating the internal invading hypha. White arrows indicate hypha that have emerged or growth through unlabelled (asterisks) cells and yellow arrows indicate intracellular parts of the hypha. Scale bars: 40 μm.

Live-cell confocal imaging demonstrated that *A. fumigatus* conidia germinate on the host surface or intracellularly during its interaction with the lung epithelia. Continuous hyphal extension of internalised spores resulted in the formation of mature hyphae which either extended into the adjacent or through many host cells. Additionally, young germlings growing within the host cells also displayed an abnormal morphology, produced more than one germ tube, extended extracellularly and subsequently invaded the adjacent cells (Figures 2A–C). Interestingly, the growing fraction of *A. fumigatus* escaping from the respiratory epithelium exhibited similar leading hyphae morphology to those growing only in cultured media.

Hyphal length was comparatively assessed on the surface and within alveolar and bronchial epithelial cells using cell culture media as control. Hyphae growing within alveolar or bronchial epithelial monolayers displayed a filament length of 23.66 and 22.08 μm, respectively. This length of the hypha was significantly lower than that observed for simultaneously inoculated conidia growing on the cell surface (Figure 1D and Supplementary Figure 1C). Notably, neither *A. fumigatus* filaments growing within nor those hyphal fractions escaping the host cell, exhibited obvious subcellular damage during early infection as shown by transmission electron microscopy imaging (Supplementary Figures 2, 3). Once hyphae escape toward the extracellular milieu, subsequent hyphal penetration to a new epithelial host cell was also observed (Figure 2C). These observations highlight the ability of *A. fumigatus* to disseminate during early stages of the colonisation of respiratory epithelia without causing significant host damage.

**FIGURE 3:**
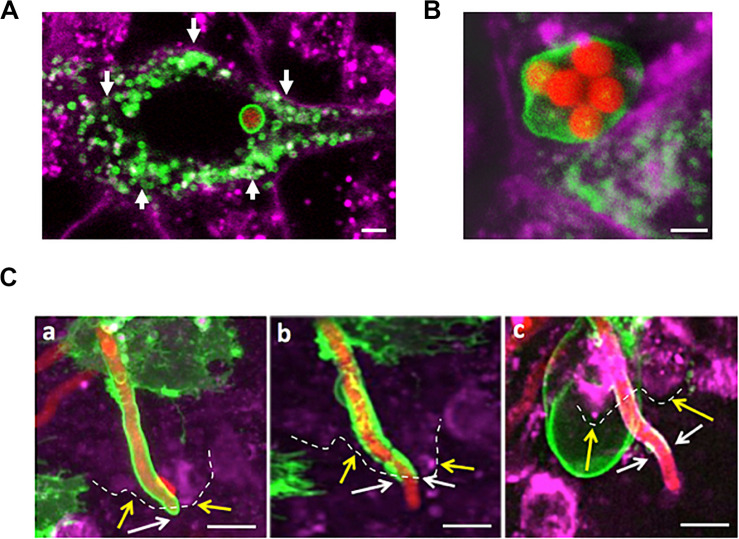
*A. fumigatus* conidia are surrounded by a phagolysosomal membrane upon internalisation, germination and during A549 escape. **(A)** Single internalised conidia surrounded by a phagolysosomal membrane. A large number of individual lysosomes are accumulated in proximity to the host cell membrane (arrows). **(B)** Multiple internalised conidia surrounded by a single phagolysosomal compartment. **(C)** Time course of escaping of an extending hyphae from the phagolysosome demonstrates this process to involve (a) stretching of the phagosome; (b) escape from the host cell and (c) escape from the phagolysosome. Yellow arrows and dashes indicate the host cytoplasmic membrane; white arrows indicate the distal part of the tip escaping from the phagolysosome (Scale bars = 5 μm). Red = *A. fumigatus* expressing cytoplasmic RFP; Green = phagolysosomal compartment stained with LAMP-1 GFP. Magenta = A549 cell plasma membrane stained with CellMask deep red.

### Internalised *A. fumigatus* Germlings Are Surrounded by a Phagolysosomal Membrane Upon A549-Host Escape

Early during the infection of the respiratory epithelium *in vitro*, internalised *A. fumigatus* spores are fused to lysosomes and acidic vesicles that inhibit the germination of the majority of the *A. fumigatus* spores (Wasylnka and Moore, 2002; Ibrahim-Granet et al., 2003; Botterel et al., 2008). Live-cell confocal imaging demonstrated that the percentage of *A. fumigatus* conidia which germinate is 5-fold decreased from 12 to 48 h in both cell lines. This reduced viability indicates that most of the internalised conidia by epithelial cells are killed at 48 h post infection and the surviving ones are able to outgrowth by hyphal extension with no obvious host cell injury (Figure 1C, Supplementary Figures 4A,B, 5). To further characterise the mechanistic of this process, internalisation, intracellular extension and escape of *A. fumigatus* germlings from the phagosome was investigated in a time-dependent manner.

After *A. fumigatus* uptake by the lung epithelium, conidia were surrounded by a single LAMP-1 expressing host membrane suggesting phagosome maturation to phagolysosome (Figure 3A). In rare occasions (<1%), a single large phagolysosome compartment surrounding several conidia was observed (Figure 3B). Persistent spores contained within phagolysosomes were able to germinate (6–9 h post-infection), and the host phagolysosomal membrane was continuously extended while surrounding the intracellular growing young germling (Figure 3C). The aforementioned phagolysosome was extended until the tip of the growing germling contacted the host plasma membrane. While the hyphae fraction that remains inside of the host cell was covered by the phagolysosomal membrane, the apical fraction of *A. fumigatus* that escaped the host cells was not further covered. This phagolysosome-plasma membrane interaction likely resulted in the migration of the host cytoplasmic membrane into the phagolysosomal membrane surrounding the intracellular growing germlings (Figure 3). This suggests that the phagolysosome and the host plasma membrane likely fuse at the point of hyphal exit in the same manner as observed during escape of other pathogens from the host (Hissa et al., 2012; Pereira et al., 2019).

**FIGURE 4:**
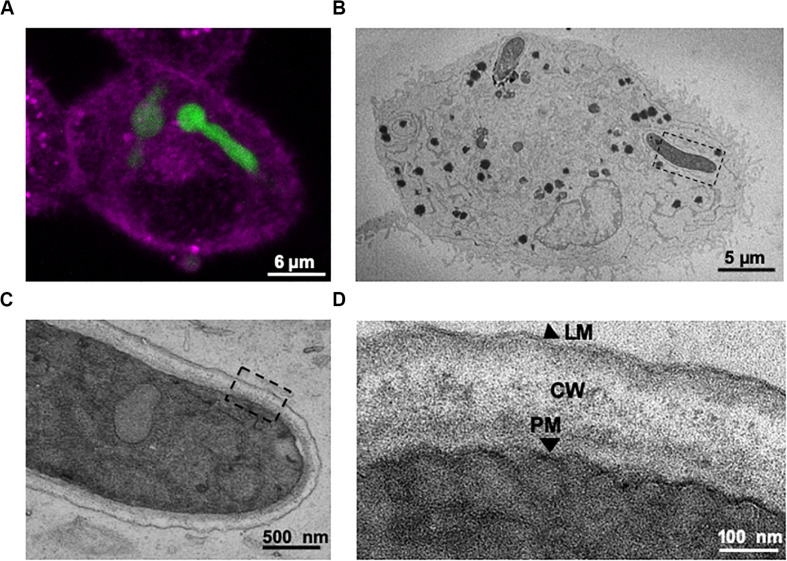
Correlative live-cell imaging and transmission electron microscopy (TEM) confirms that internalised *A. fumigatus* growing germlings are engulfed within the lung epithelium phagosome. **(A)** Confocal live-cell imaging of an internalised germling by A549 epithelial cells. **(B)** TEM image of a cross-section of the same infected A549 cell. **(C)** Magnification of the tip of the internalised germling. **(D)** Sections of the germling cell wall, PM: *A. fumigatus* plasma membrane; CW: *A. fumigatus* cell wall; LM: host phagolysosomal membrane. Note the host’s lipid bilayer that surrounds *A. fumigatus* within A549 host cells shown in panel **(D)**.

Transmission electron microscopy (TEM) was performed upon A549 alveolar epithelial cells during *A. fumigatus* challenge to confirm the results from confocal imaging. 3-D datasets (Figure 4A) of A549 epithelial cells containing internalised *A. fumigatus* germlings were created followed by TEM analysis (Figure 4B). On an ultrastructural level, *A. fumigatus* germlings were tightly surrounded by a host’s lipid-bilayer membrane prior to escape (Figures 4C,D), which confirmed our initial live-cell confocal imaging observations (Figure 3). Notably, this presumably LAMP-1 phagolysosomal-associated membrane was intact during its interaction with *A. fumigatus*. Upon interaction of the extending phagosome covering the growing hyphal tip with the host plasma membrane, a protrusion of plasma membrane covering the apical area of the hyphae was noticed which might play a role in preventing host’s death (Supplementary Figure 3).

### The Alveolar and Bronchial Plasma Membrane Fuses With the Phagosomal Membrane After *A. fumigatus* Escape

**FIGURE 5:**
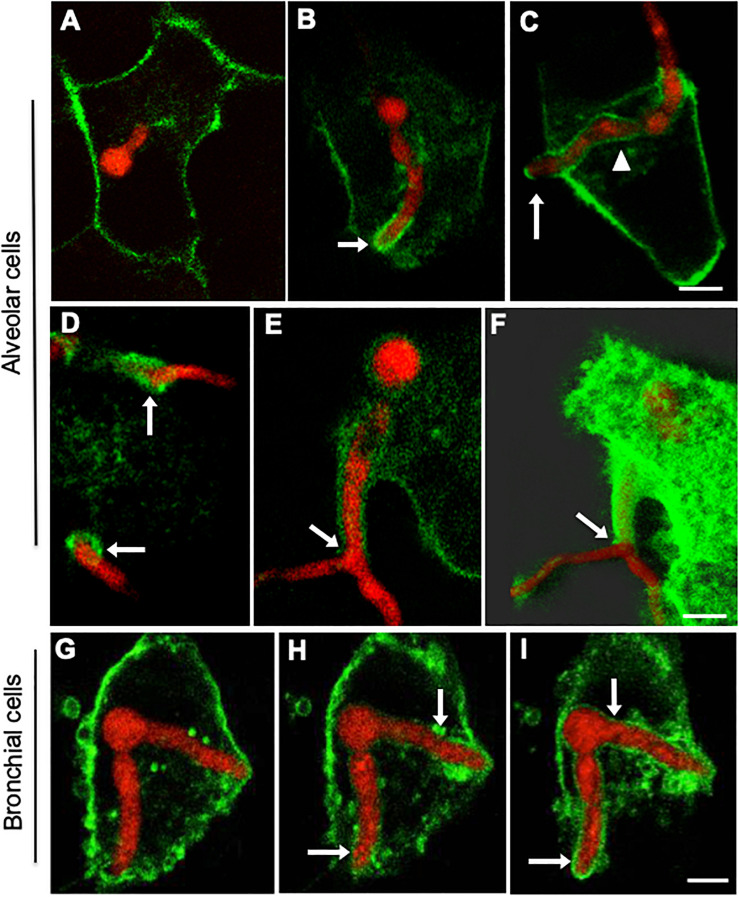
Internalised *A. fumigatus* cells are surrounded by the host’s plasma membrane upon escape of alveolar and bronchial epithelial cells. Confocal live-cell imaging shows *A. fumigatus* expressing cytoplasmic RFP within A549 and 16HBE epithelial cells expressing GFP in the plasma membrane. **(A)** Internalised germling before reaching the host plasma membrane. **(B)** The tip (arrow) of the internalised germlings is surrounded by host plasma membrane during early interaction. **(C)** After late interaction of the internalised germling tip, the host plasma membrane surrounds the basal part of the germling (arrowhead). **(D)** Host plasma membrane is accumulated in the regions of hyphal escape (arrows). **(E)** Host plasma membrane surrounds an extension of the escaping germling. **(F)** Maximal projection shown in panel **(E)**. **(G–I)** 16HBE plasma membrane (arrows) surrounds internalised *A. fumigatus* germlings upon escape. Scale bars = 5 μm.

We previously observed that the plasma membrane-specific dye (CellMask Deep Red) was able to stain the epithelial cell plasma membrane but also partially labelled the membrane surrounding the portion of the germling remaining within the host during escape (Figure 3). However, at this stage we were unable to discriminate whether this dye was either staining the plasma membrane of the host or *A. fumigatus*. To overcome this problem, A549 and 16HBE epithelial cells were transfected with a GFP plasma membrane specific marker for mammalian cells (GFP-myristolyation/palmitoylation sequence from Lck tyrosine kinase) and monitored during *A. fumigatus* infection as previously described. Uninfected epithelial cells showed a homogeneous GFP signal throughout the epithelial cell plasma membrane with no evidence of transfection toxicity (data not shown). During *A. fumigatus* challenge experiments, the lck-GFP marker did not label the fungal plasma membrane (Figure 5), despite germlings being enclosed within phagolysosomes (Figure 3). Interestingly, an accumulation of lck-GFP surrounding extending *A. fumigatus* hyphal tips was evident in both alveolar and bronchial epithelial cells prior to hyphal escape (Figures 5B–H; Supplementary Video 3). As hyphal escape progressed, the lck-GFP plasma membrane signal slowly migrated into the host lipid bilayer membrane surrounding both growing germlings and basal conidia with no obvious fungal cytological damage (Figure 5C). Remarkably, an accumulation of strained host plasma membrane signal was observed around the points of fungal escape in agreement with the TEM experiments described above (Figures 5D, 4 and Supplementary Figure 3). Maximal projections of the same infected epithelial cells and ultrastructural analysis showed that the host’s plasma membrane was sealed upon hyphal escape with no loss of cell contents (Figure 5F, Supplementary Figure 5) presumably by fusion with the phagolysosomal membrane (Figures 3D,E).

### *A. fumigatus* Mature Germlings Are Surrounded by a Non-acidic Compartment

One of the early features of phagosome maturation is acidification. Low pH directly affects many pathogens and provides optimal pH for maximal activity of many hydrolytic enzymes (Uribe-Querol and Rosales, 2017). To determine whether the observed fungal germination and hyphal growth within the host epithelium was linked to reduced *A. fumigatus*-containing phagosome acidification, confluent alveolar (A549) and bronchial (16HBE) epithelia monolayers were labelled with the pH sensor LysoTracker Red^™^ DND-99. The progress of acidification within conidium-containing phagosomes was tracked for 2 h.

**FIGURE 6:**
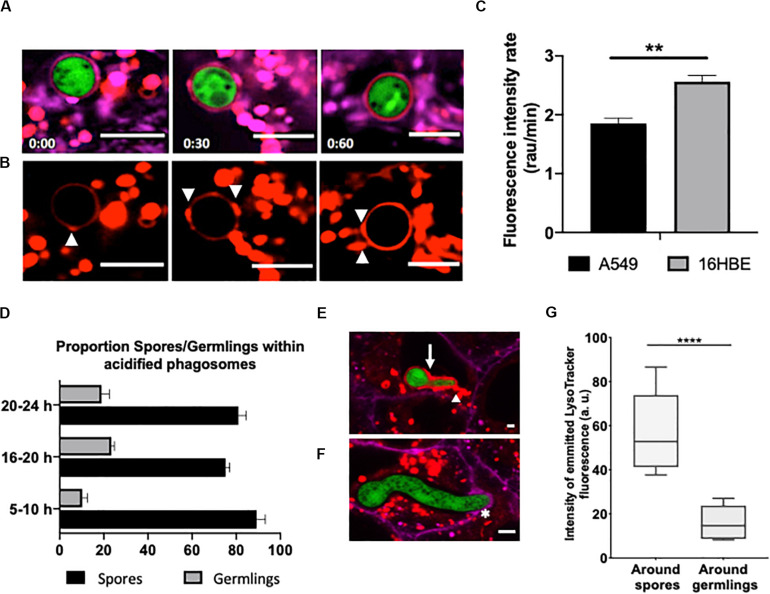
Escaping *A. fumigatus* germlings are surrounded by a non-acidic phagosomal compartment. Alveolar monolayers were challenged with *A. fumigatus* (green) for 18 h at 37°C, 5% CO_2_. Following incubation times, cells were stained with Cell Mask Deep Red (magenta) and LysoTracker (red). **(A)** Time lapse (min) of an internalised spore surrounded by an acidic phagosome. **(B)** LysoTracker channel shown in A. Arrowheads highlight the site of lysosome fusion with the phagosome. **(C)** Acidification rate of spores internalised by either alveolar or bronchial cells. r.a.u estates for relative abundance units. **(D)** Time-lapse quantification of the percentage of germlings surrounded by acidified and non-acidified phagosomes. **(E)** Merged images of an internalised young germling. The arrow highlights the acidic compartment that surrounds *A. fumigatus*. The arrowhead highlights the fusion site of the acidic compartments. **(F)** Merged images of an internalised mature germling. The asterisk highlights the initial interaction between the mature germling tip and the protrusion of the host plasma membrane. Note there is no fusion of acidic compartments surrounding *A. fumigatus*. **(G)** Relative fluorescence of LysoTracker in internalised spores and germlings. Measurements were performed three times on three biological samples (average ± standard deviation [SD]) (**P < 0.01 and ****P < 0.0001). Scale bars: 5 μm.

After engulfment of *A. fumigatus*, spores were co-localised within phagosomes which were acidified on a time-dependent manner by the addition of small acidic vesicles at discrete sites around the conidium (Figures 6A,B). This acidification process was observed to commence 5 min after engulfment. Overall, the increase in acidification rate was significantly higher in *A. fumigatus* containing phagosomes of 16HBE bronchial epithelial cells compared to A549 alveolar epithelial cells, which suggest improved fungal clearance capability of the bronchial epithelium (Figure 6C). Only one third of the germlings were surrounded by acidic phagosome after 24 h of infection while spores which were mainly contained within acidified phagosomes (Figures 6D–F). Overall, phagosomes containing spores displayed ∼4-fold higher fluorescence intensity compared to phagosomes containing germlings (Figure 6G, Supplementary Figure 6). Remarkably, phagosome acidification was not observed in the recipient cell after escape (Supplementary Figure 5).

These results suggest that successful phagosome acidification prevents spore germination within the phagosome. Although a potential alternative would be that the phagosomal membrane loses integrity and reduces phagosome efficacy, loss of integrity or leakage of acidic contents into the cytoplasm were not observed in this study.

### Host Plasma Compartmentalisation of *A. fumigatus* Hyphae Occurs During Invasion of Neighbouring Naive Host Cells

Based on our previous observation of the capability of *A. fumigatus* hyphae to grow through an epithelial cell layer without causing cell injury (Figure 2), we hypothesised that lack of host cell injury could be explained by failure of the hypha to rupture the host plasma membrane. To test this, A549 expressing a GFP labelled plasma membrane were challenged with *A. fumigatus* spores and visualised over 24 h.

**FIGURE 7:**
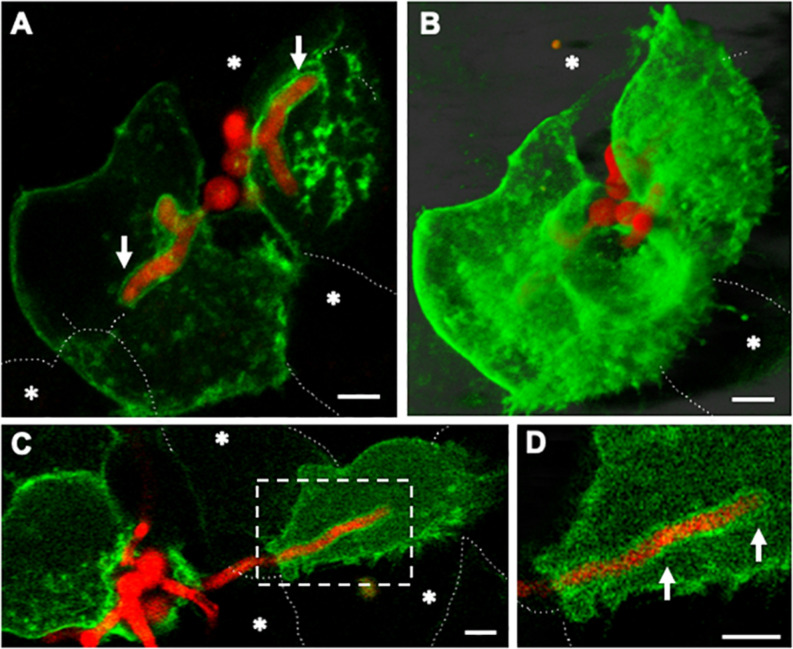
Host plasma membrane compartmentalisation of *A. fumigatus* hyphae occurs upon invasion of naïve lung epithelial neighbouring cells. Confocal live-cell imaging of *A. fumigatus* expressing cytoplasmic RFP within a GFP-plasma membrane transfected A549 epithelial cell line at 37°C, 5% CO_2_. **(A)** Germ-tubes of internalised spores extend toward adjacent cells. **(B)** Maximal projection of the same cells shown in A. **(C)** Upon escape of an epithelial host the apical zone of an invading leading hyphae is surrounded by the plasma membrane of a naïve host cell. **(D)** Zoom in of the apical region of the hypha shown in **(A)**. White arrows indicate the plasma membrane of the new host. Asterisks highlight epithelial cells that were not transfected. Scale bars: 5 μm.

Escaping hyphae were able to either grow on the upper surface of the host cells, or toward neighbouring uninfected lung epithelial cells. The portion of hyphae extending into uninfected neighbouring cells was surrounded by the plasma membrane of the recipient cell (Figure 7). Epithelial cell injury or death in the fungal recipient epithelial cells was not observed. Interestingly, the apical segment of *A. fumigatus* hyphae growing in neighbouring uninfected epithelial cells was also morphologically unaffected. These results show that *A. fumigatus* hyphae that grow through adjacent epithelial cells are also surrounded by plasma membrane of the new host cell (Fernandes et al., 2018).

**FIGURE 8:**
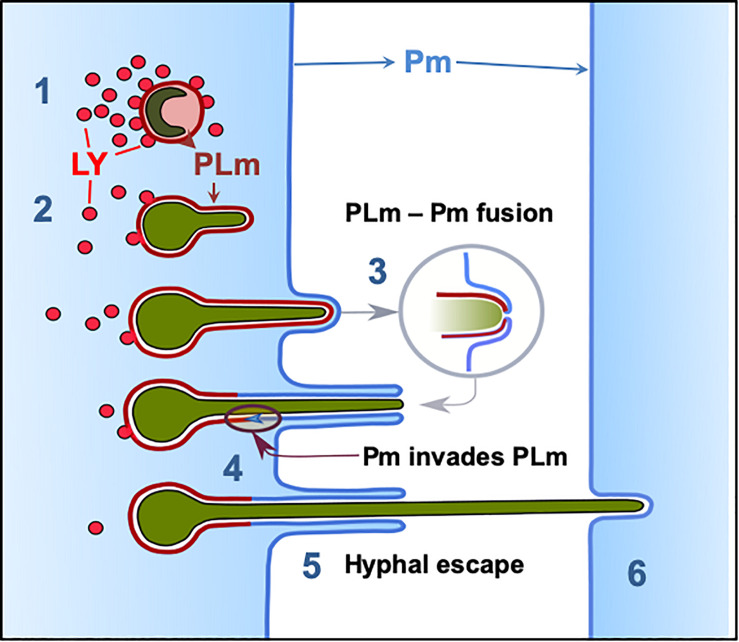
Phagolysosome–Plasma membrane dynamics during *A. fumigatus* escape. (1) Most *A. fumigatus* conidia are killed by acidification of phagosomes and lysosomal fusion, (2) In some circumstances lysosome recruitment to the phagosome containing *A. fumigatus* conidia does not inhibit fungal growth and acidification does not occur allowing conidial germination. (3) The PLm continuously extends while surrounding the intracellular growing germling until the PLm contacts the plasma membrane. (4) The PLm and Pm fuse and (5) the host plasma membrane migrates into the phagolysosome membrane facilitating hyphal escape. (6) Hyphae then proceed to penetrate further host cells without rupture of the plasma membrane. LY: lysosome, PL: phagolysosome, PLm; phagolysosome membrane, Pm: plasma membrane.

Altogether, our results suggest that phagosome maturation failure facilitates *Aspergillus* germination and escape in a non-lytic manner. Upon hyphal escape, the phagosome membrane which can be at different maturation stages and cytoplasmic membrane are likely fused, sealing the host intracellular environment and avoiding the host cell death (Figure 8). Neighbouring cell-penetrating hyphae were also surrounded by recipient host cell plasma membrane.

## Discussion

The efficiency of alveolar macrophages in ingesting and killing *A. fumigatus* spores early after infection has hidden the potential role of the most abundant host cells in the lung, the airway epithelium (Crapo et al., 1982; Latgé, 1999; Luther et al., 2008; Croft et al., 2016). However, the relevance of uptake by epithelial cells of metabolically active germinating *A. fumigatus* early after infection remains unclear.

The development of mature hyphae requires *A. fumigatus* spore germination. This process depends on several factors including pH, temperature and the presence of stressors such as antifungal agents (Araujo and Rodrigues, 2004; Moreno-Velásquez et al., 2017). In previous studies (Escobar et al., 2016; Richard et al., 2018), impaired germination of *A. fumigatus* spores has been reported on alveolar, bronchial and primary lung epithelial cells in agreement with our results. However, our data further show that germination of internalised *A. fumigatus* conidia is one hour delayed compared to those growing on the surface of the respiratory epithelia (Wasylnka and Moore, 2002; Escobar et al., 2016) in the same manner as it is after phagocytosis by macrophages (Schaffner et al., 1983; Van Waeyenberghe et al., 2012). This delay in spore germination within epithelial cells is not surprising considering the stressful conditions within phagosomes and phagolysosomes (Heinekamp et al., 2015). Furthermore, we observed that the cellular environment of human host induced an aberrant morphology and growth of internalised *A. fumigatus* young germlings, such as reduced hyphal extension, increased branching, and a large number of vacuoles. Changes in fungal morphology within host cells characterised by the generation of new hyphal branches have been previously described as a fungal mechanism to facilitate invasive growth (Ellett et al., 2017). No host cell injury was noticed after germination and growth of internalised *A. fumigatus*, even when individual epithelial cells were infected with several germlings, as reported for both, professional and non-professional phagocytes (Paris et al., 1997; Rosowski et al., 2018).

Here, we observed that acidification was four-fold reduced in phagosomes containing growing germlings compared to those containing spores. Inability of macrophages to sustain acidification has been reported as a mechanism for bacteria replication and escape from the host environment (Jubrail et al., 2015). Additionally, increased fungal size leads to reduced phagosome and phagolysosome acidification of engulfed *Candida albicans* and *Cryptococcus neoformans* cells by macrophages while promoting fungal escape and host integrity (DeLeon-Rodriguez and Casadevall, 2016; Westman et al., 2018). A similar escaping mechanism has been described for *A. fumigatus* during epithelial cell infection and confirmed by us in this study (Wasylnka and Moore, 2002; Filler and Sheppard, 2006; Croft et al., 2016; Escobar et al., 2016). In some circumstances we observed the capacity of lung epithelial cells to form large phagolysosomes, which contained several ingested conidia as previously observed in macrophages (Alpuche-Aranda et al., 1994; Luther et al., 2008).

Several pathogenic fungi are able to invade mammalian host cells by a species-specific lytic and non-lytic egress mechanisms (Káposzta et al., 1999; Filler and Sheppard, 2006; Escobar et al., 2016). For example, *C. albicans* can form germ tubes within phagolysosomes facilitating fungal escape and macrophage killing (Káposzta et al., 1999). Similarly, phagocytosis of *C. neoformans* by macrophages results in aberrant lysosomal trafficking, pathogen release and death (Feldmesser et al., 2001). In *A. fumigatus*, internalised conidia are trafficked via the late endosomes/lysosomes route (Botterel et al., 2008). Some studies have shown that a small population of conidia can germinate and escape the host cells with no obvious evidence of host cell death (Wasylnka and Moore, 2002; Escobar et al., 2016). To further characterise this process using live-cell imaging we showed that hyphal tips of *A. fumigatus* are able to emerge either to the extracellular milieu or towards neighbouring epithelial cells. These escaping filaments displayed unaffected apical morphology compared to the internalised fragments and only a small proportion of host cells were detached or killed.

With a view to further identifying the mechanism underlying *A. fumigatus* escape, a combinatorial approach using live-cell confocal imaging and TEM was performed. Our data demonstrated a transient interaction between the mature phagosome and plasma membrane of the host upon *A. fumigatus* escape which reduces host injury (DeHart et al., 1997; Zhang et al., 2005). In order to escape from the host antimicrobial response, *A. fumigatus* conidia within mature phagosomes dysregulate several cellular processes such as Lamp1 and vATPase-driven acidification, Rab5 and Vamp8-dependent endocytic trafficking or cathepsin Z expression (Schmidt et al., 2018). Additionally, *A. fumigatus* is able to control some host cellular responses by impairing phagosome-lysosome fusion, inhibiting apoptosis or preventing the oxidative stress responses of lung epithelial cells (Jahn et al., 2002; Berkova et al., 2006; Féménia et al., 2009; Gomez et al., 2010; Oosthuizen et al., 2011; Thywißen et al., 2011). Therefore, a process combining reduction of phagosome maturation, resistance to digestion and hyphal escape via a non-lytic mechanism would allow *A. fumigatus* to survive and escape without causing significant cellular damage to the human host cell (Figure 8). Given that only a fraction of spores survive, we suggest that some spore subpopulations are able to adapt to conditions within the phagolysosome and successfully germinate leading to ramification of hyphae through the living epithelium, thus facilitating the development of invasive or chronic conditions (Grant and Hung, 2013; Ballard et al., 2018).

In this work, we highlighted that internalised *A. fumigatus* does not directly interact with the cytosol of lung epithelial cells. Additionally, penetration of intracellular hyphae growing out of phagolysosomal compartments in neighboring cells does not break the host plasma membrane. It is possible that *A. fumigatus* hyphae ramifying through epithelial cells in this manner are able to actively acquire nutrients from the host in the same manner as that displayed by haustoria in biotrophic plant-fungal interactions. Host plasma membrane coating of fungi allows host receptors to be triggered but limits the ability of immune responses particularly macrophages and neutrophils to reach the fungus (Berkova et al., 2006). Further molecular studies are required to determine whether this is a defense mechanism governed by the host or controlled by the fungus.

## Data Availability Statement

The raw data supporting the conclusions of this article will be made available by the authors, without undue reservation.

## Author Contributions

CS, SM-V, and NB-G performed the experiments and analysed the data. CS, SM-V, SG, NR, and PB conceived, drafted and edited the manuscript. All authors have given approval to the final version of the manuscript.

## Conflict of Interest

The authors declare that the research was conducted in the absence of any commercial or financial relationships that could be construed as a potential conflict of interest.

## Footnotes

1 https://fiji.sc/
