# Supplementary material for: Phagolysosomal Survival Enables Non-lytic Hyphal Escape and Ramification Through Lung Epithelium During Aspergillus fumigatus Infection
Source: Front Microbiol. 2020 Aug 20;11:1955. doi: 10.3389/fmicb.2020.01955 (PMC7468521; doi:10.3389/fmicb.2020.01955)
Supplement: Supplementary file 1 [file Image_1.pdf]

Supplementary figure 1

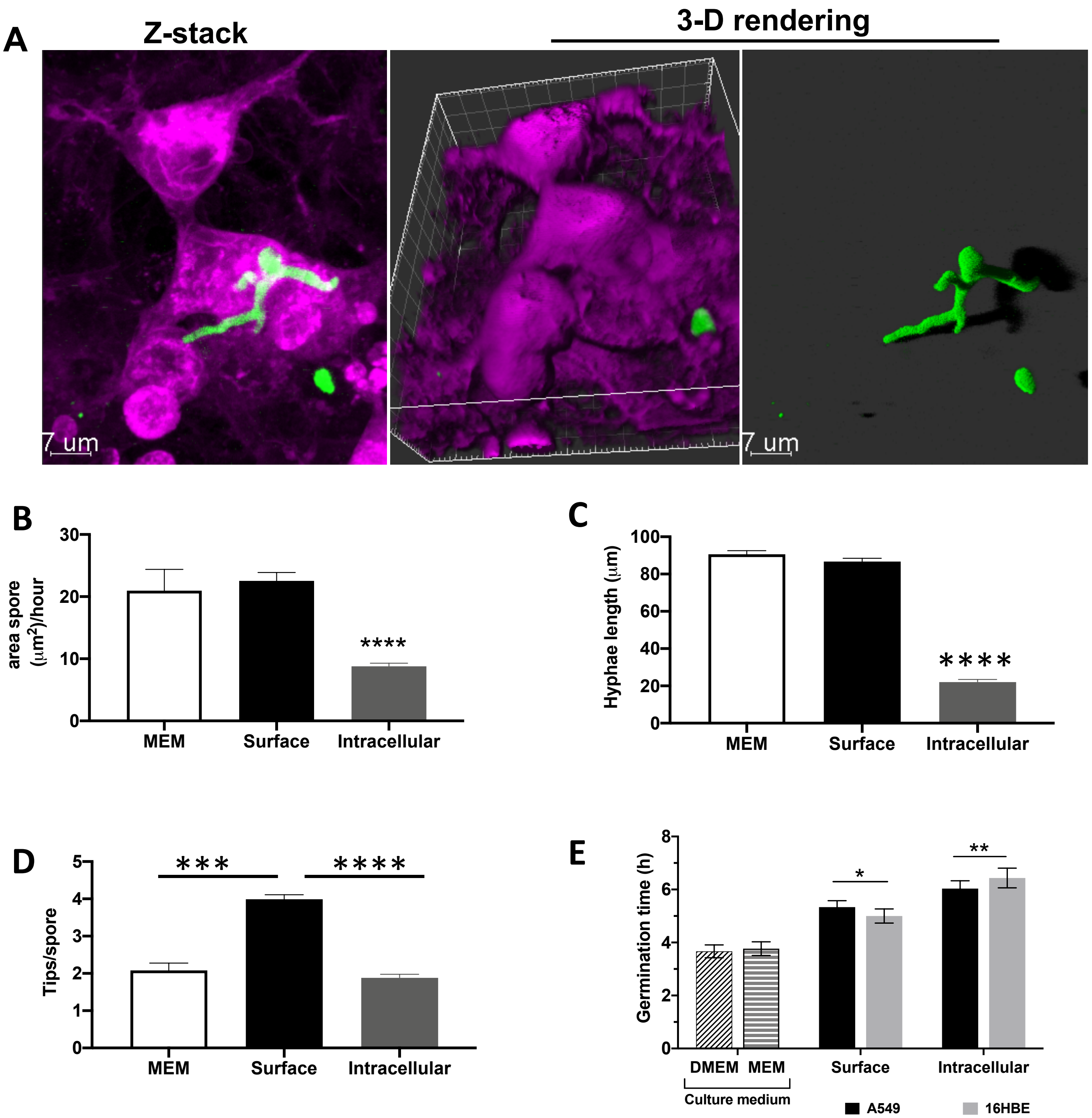

FIGURE S1. Cellular morphogenesis of *A. fumigatus* upon infection of 16HBE bronchial epithelial cells. (A Live-cell imaging of 16HBE bronchial epithelial cells (magenta) infected with hyperbranched *A. fumigatus* expressing cytoplasmic GFP for 18 h at 37 °C, 5 % CO_2. *A. fumigatus* **(B)** *g*ermination rate, **(C)** Hyphae extension and **(D)** Branching in the presence or absence of alveolar cells. Measurements were performed three biological and technical replicates (average ± standard deviation [SD]). * $P < 0.05$; ** $P < 0.01$; *** $P < 0.001$; **** $P < 0.0001$. **(B)** *g*ermination rate

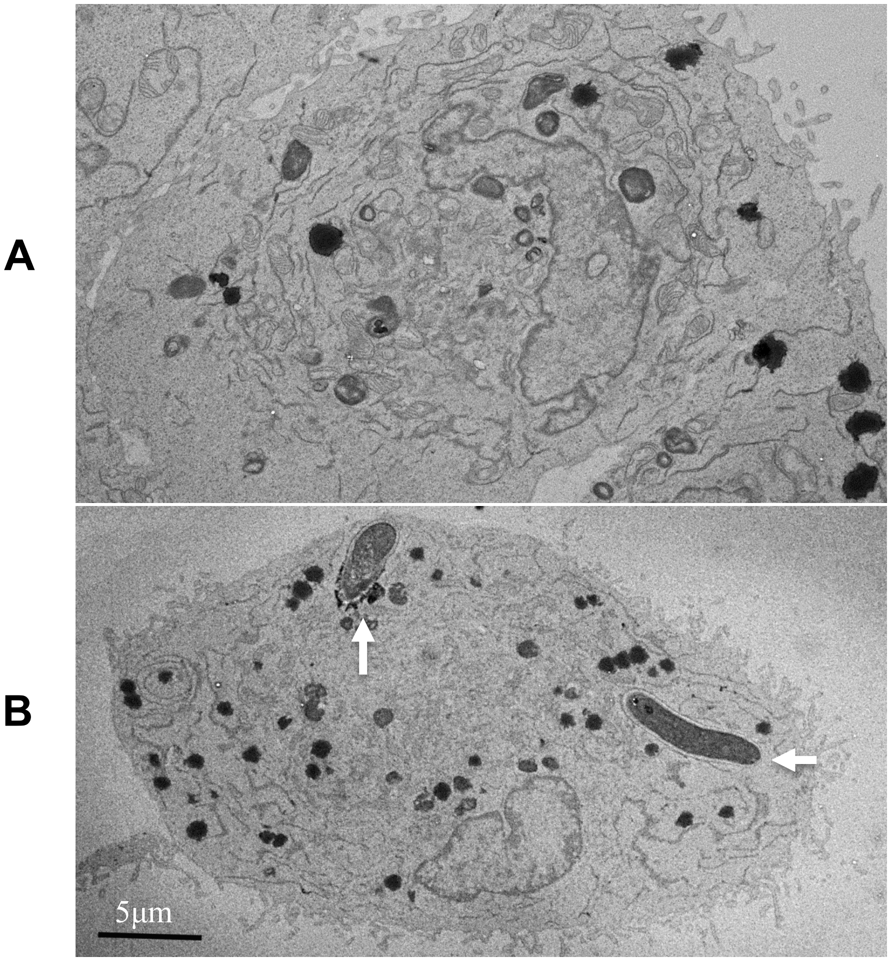

FIGURE S2. Transmission electron micrographs of the cellular architecture of human alveolar cells. Alveolar A549 monolayers were challenged with *A. fumigatus* for 18 h in DMEM at 37 °C, 5 % CO_2. **(A)** An uninfected alveolar cell **(B)** An alveolar cell infected by *A. fumigatus* (white arrows). Note that the infected alveolar cell is the same as used in Fig. 4.

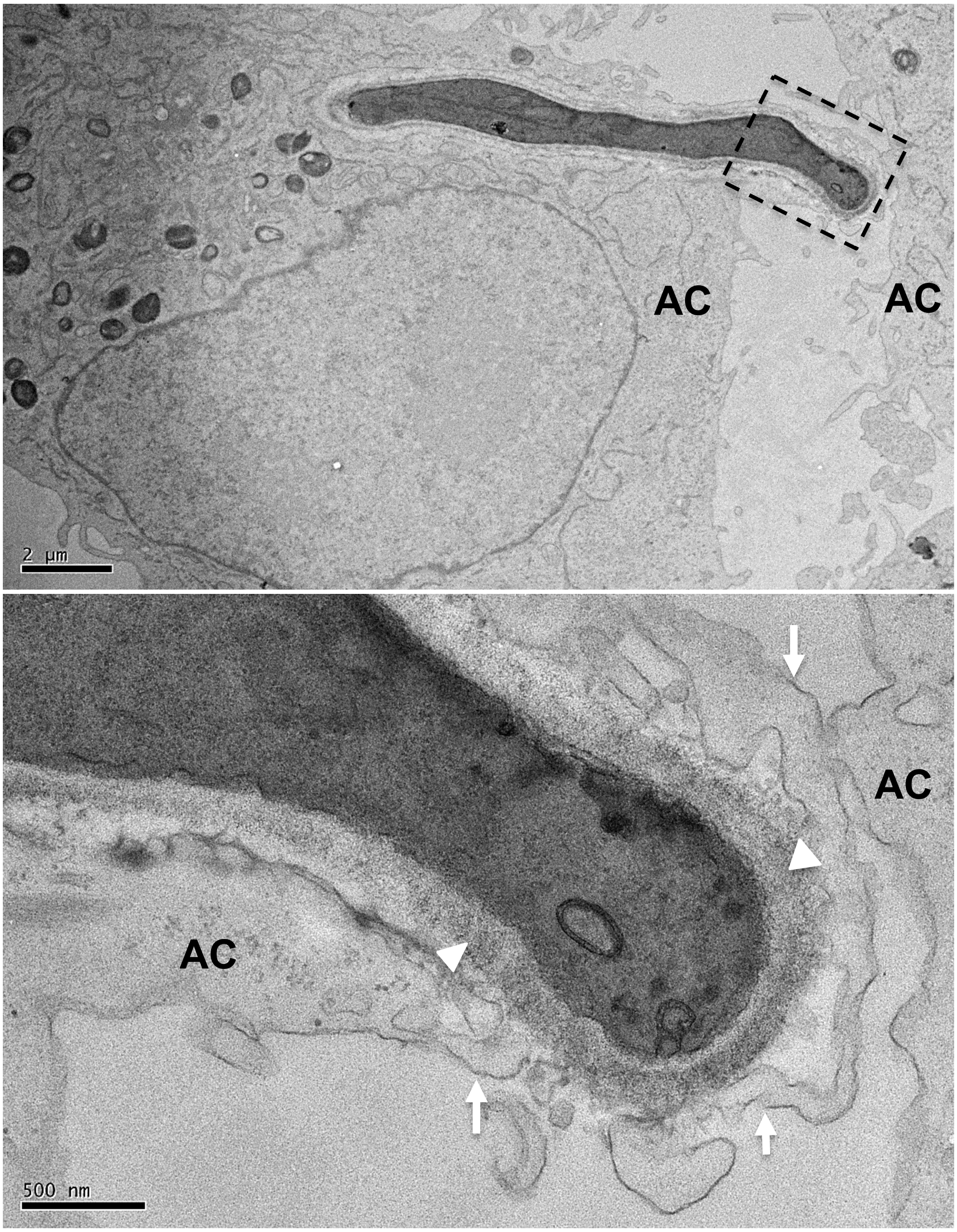

FIGURE S3. Internalized hyphae of *A. fumigatus* cause the protrusion of the host plasma membrane upon escape. Transmission electron micrographs of an internalized *A. fumigatus* germling escaping from a host alveolar cell. Nystatin treated alveolar A549 monolayers were challenged with *A. fumigatus* for 18 h in DMEM at 37 °C, 5 % CO_2. **(A)** Low magnification of a escaping hypha. **(B**) Zoom in shown in A of the same escaping germling. Note the protrusion of host plasma membrane (white arrows) that covers the apical area of the *A. fumigatus* tip upon escape. Arrowheads highlight the cell wall of *A. fumigatus*. AC: alveolar cell.

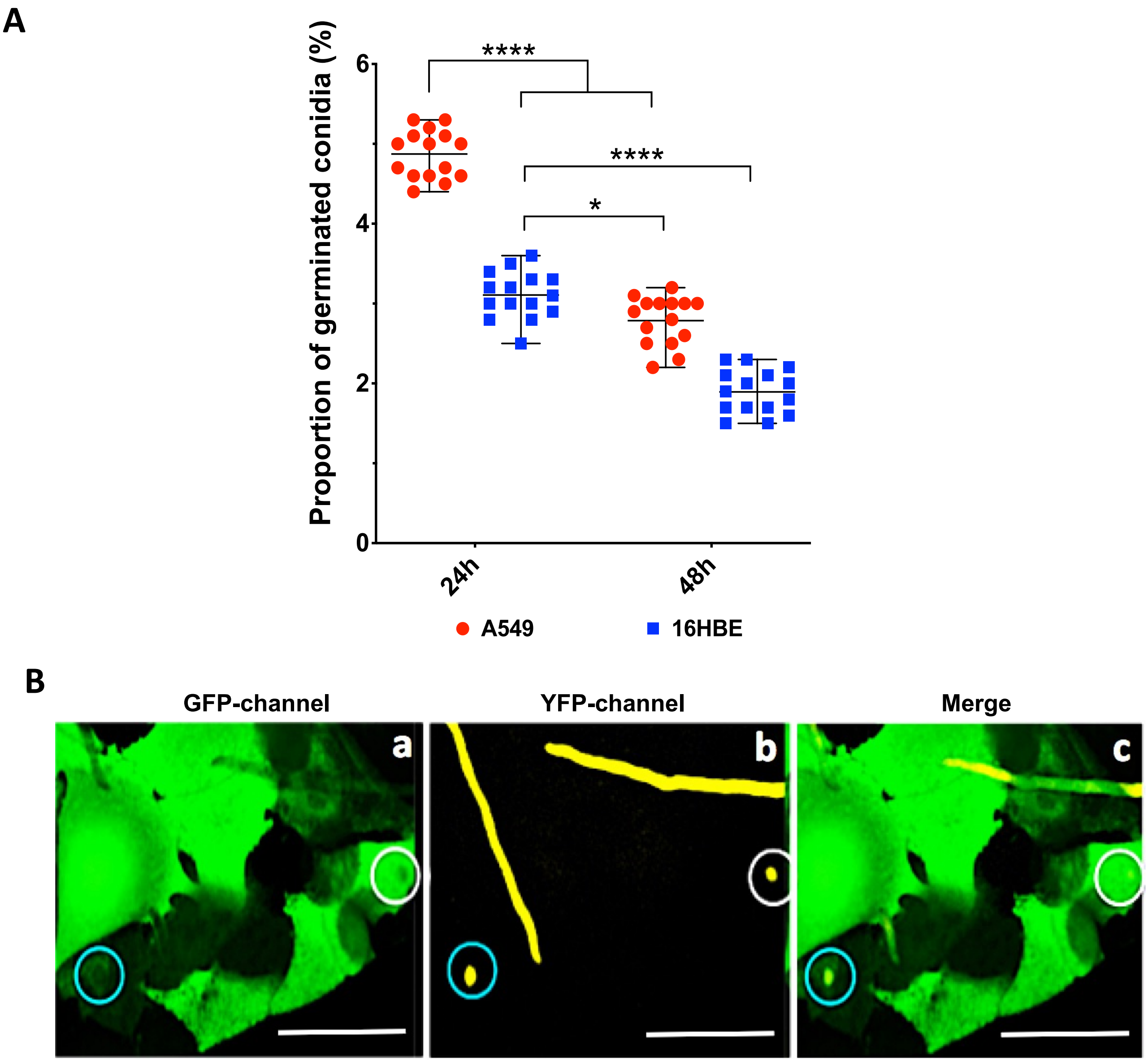

FIGURE S4. Persistence of *A. fumigatus* conidia within A549 epithelial cells. A) About 5% of *A. fumigatus* conidia remains latent within A549, which was significantly higher than in 16HBE (3.11%) 24 h post-challenge. Persistent conidia was reduced in both cell lines (2.79% and 1.89%). Differences were compared using two-way ANOVA (**P* <0.02, *****P* <0.0001). **B)** 3-D confocal Z-stack, **a)** cytosolic-GFP A549-epithelial cells (green), **b)** cytosolic-YFP (yellow) of *A. fumigatus* (gift from Sven Krappmann), and **c)** a merge of both channels. A small proportion of *A. fumigatus* internalized conidia had the ability to remain latent without germination for more than 48 h. (63x). Scale Bar = 40 μm.

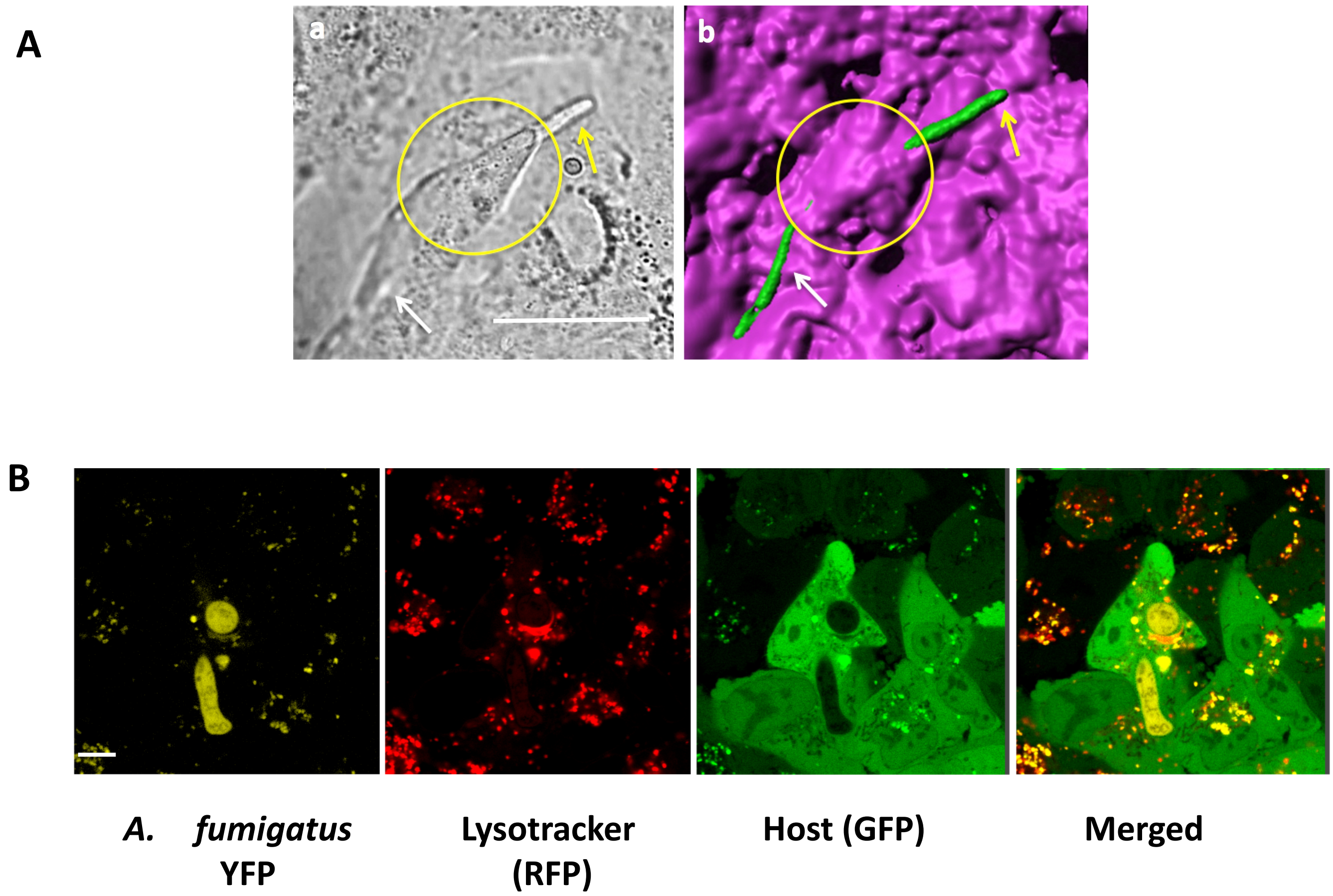

FIGURE S5. A) 3-D confocal image of a hypha (green) that has germinated within the epithelial cell (magenta) and emerged extracellularly (indicated by white arrow) and then penetrated the adjacent cell (indicated by a yellow circle) and exited again (indicated by a yellow arrow) 18 h post-inoculation. **B)** 3-D confocal stack demonstrate an *A. fumigatus* germling (yellow) were localized within GFP-cytosolic A549 cell (green) that germinated intracellularly and extended its hyphae to escape without causing epithelial cell damage. Red shows acidification failure during hyphae escape (63x). Scale Bar = 5 μm.

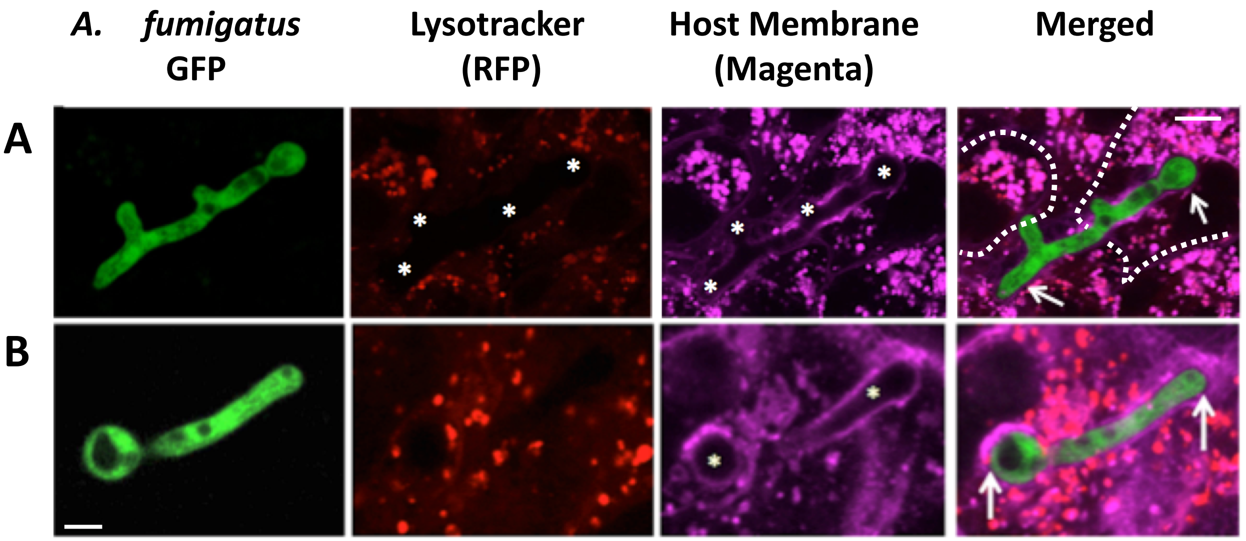

Supplementary Figure 6: (A) Germinated *A. fumigatus* spores within un-acidic phagosomes, which grow (arrows), started to branch, and invading the adjacent cells (dash). **(B)** Lack or reduction of phagosome acidification facilitated *A. fumigatus* conidia germination and growth 18h post-inoculation. Asterisk represent points of active growth. Asterisks represent fungal regions of active growth (63x) Scale Bar = 5 µm.
