# Supplementary material for: Phagolysosomal Survival Enables Non-lytic Hyphal Escape and Ramification Through Lung Epithelium During Aspergillus fumigatus Infection
Source: Front Microbiol. 2020 Aug 20;11:1955. doi: 10.3389/fmicb.2020.01955 (PMC7468521; doi:10.3389/fmicb.2020.01955)
Supplement: Supplementary file 2 [file Table_1.docx]

**Video Legends**

**Supplementary Video 1. Cellular morphogenesis of internalized *A. fumigatus* by epithelial cells**. 3D-rendering of GFP *A. fumigatus* within A549 epithelial cells at 18 h post infection. Epithelial monolayer (magenta) was stained with Cell Mask. Note that despite the extension of internalized *A. fumigatus*, the integrity of the monolayer is significantly unaffected.

**Supplementary Video 2. *A. fumigatus* morphogenesis is impaired upon intracellular infection of 16HBE bronchial cells**. 16HBE monolayers were challenged with *A. fumigatus* constitutively expressing cytoplasmic GFP in MEM at 37 °C, 5% CO_2_ for 18 h. Epithelia monolayer was stained with cell mask deep red (magenta). Note that despite the extension of internalized *A. fumigatus*, the integrity of the monolayers is significantly unaffected.

**Supplementary Video 3. Host plasma membrane surrounds internalized *A. fumigatus* upon escape.** 16HBE bronchial monolayers were transfected with a plasma membrane marker (GFP-myristolyation/palmitoylation) and *A. fumigatus* constitutively expressing cytoplasmic RFP and cultured in MEM at 37 °C, 5% CO_2_ for 18 h.
